# Microstructural neural correlates of maximal grip strength in autistic children: the role of the cortico-cerebellar network and attention-deficit/hyperactivity disorder features

**DOI:** 10.3389/fnint.2024.1359099

**Published:** 2024-05-14

**Authors:** Olivia Surgent, Jose Guerrero-Gonzalez, Douglas C. Dean, Nagesh Adluru, Gregory R. Kirk, Steven R. Kecskemeti, Andrew L. Alexander, James J. Li, Brittany G. Travers

**Affiliations:** ^1^Waisman Center, University of Wisconsin-Madison, Madison, WI, United States; ^2^Neuroscience Training Program, University of Wisconsin-Madison, Madison, WI, United States; ^3^Department of Medical Physics, University of Wisconsin-Madison, Madison, WI, United States; ^4^Department of Pediatrics, University of Wisconsin-Madison, Madison, WI, United States; ^5^Department of Radiology, University of Wisconsin-Madison, Madison, WI, United States; ^6^Department of Psychiatry, University of Wisconsin-Madison, Madison, WI, United States; ^7^Psychology Department, University of Wisconsin-Madison, Madison, WI, United States; ^8^Occupational Therapy Program in the Department of Kinesiology, University of Wisconsin-Madison, Madison, WI, United States

**Keywords:** autism, ADHD, grip strength, diffusion imaging, structural imaging, cerebellum, motor, proprioception

## Abstract

**Introduction:**

Maximal grip strength, a measure of how much force a person’s hand can generate when squeezing an object, may be an effective method for understanding potential neurobiological differences during motor tasks. Grip strength in autistic individuals may be of particular interest due to its unique developmental trajectory. While autism-specific differences in grip-brain relationships have been found in adult populations, it is possible that such differences in grip-brain relationships may be present at earlier ages when grip strength is behaviorally similar in autistic and non-autistic groups. Further, such neural differences may lead to the later emergence of diagnostic-group grip differences in adolescence. The present study sought to examine this possibility, while also examining if grip strength could elucidate the neuro-motor sources of phenotypic heterogeneity commonly observed within autism.

**Methods:**

Using high resolution, multi-shell diffusion, and quantitative R1 relaxometry imaging, this study examined how variations in key sensorimotor-related white matter pathways of the proprioception input, lateral grasping, cortico-cerebellar, and corticospinal networks were associated with individual variations in grip strength in 68 autistic children and 70 non-autistic (neurotypical) children (6–11 years-old).

**Results:**

In both groups, results indicated that stronger grip strength was associated with higher proprioceptive input, lateral grasping, and corticospinal (but not cortico-cerebellar modification) fractional anisotropy and R1, indirect measures concordant with stronger microstructural coherence and increased myelination. Diagnostic group differences in these grip-brain relationships were not observed, but the autistic group exhibited more variability particularly in the cortico-cerebellar modification indices. An examination into the variability within the autistic group revealed that attention-deficit/hyperactivity disorder (ADHD) features moderated the relationships between grip strength and both fractional anisotropy and R1 relaxometry in the premotor-primary motor tract of the lateral grasping network and the cortico-cerebellar network tracts. Specifically, in autistic children with elevated ADHD features (60% of the autistic group) stronger grip strength was related to higher fractional anisotropy and R1 of the cerebellar modification network (stronger microstructural coherence and more myelin), whereas the opposite relationship was observed in autistic children with reduced ADHD features.

**Discussion:**

Together, this work suggests that while the foundational elements of grip strength are similar across school-aged autistic and non-autistic children, neural mechanisms of grip strength within autistic children may additionally depend on the presence of ADHD features. Specifically, stronger, more coherent connections of the cerebellar modification network, which is thought to play a role in refining and optimizing motor commands, may lead to stronger grip in children with more ADHD features, weaker grip in children with fewer ADHD features, and no difference in grip in non-autistic children. While future research is needed to understand if these findings extend to other motor tasks beyond grip strength, these results have implications for understanding the biological basis of neuromotor control in autistic children and emphasize the importance of assessing co-occurring conditions when evaluating brain-behavior relationships in autism.

## 1 Introduction

As many as 88% of autistic children experience motor challenges (Bhat, 2020) that can meaningfully impact participation in activities of daily life (Jasmin et al., 2009; Travers et al., 2017) and overall quality of life (Hedgecock et al., 2018). Although considerable heterogeneity exists among the motor profiles of autistic individuals (Surgent et al., 2021), group-level differences in motor behavior are well documented in areas including fine motor (Libertus et al., 2014; Anzulewicz et al., 2016), gross motor (Bhat et al., 2011; Ament et al., 2015; Sumner et al., 2016), balance (Ardalan et al., 2019; Surgent et al., 2021), coordination (Fournier et al., 2010), hand and arm movements for pointing (Torres et al., 2013; Wu et al., 2018), and hand grip (Kern et al., 2013; Travers et al., 2015, 2017). Diagnostic-based differences in hand grip may be particularly informative as recent work suggests that maximal grip strength is representative of how refined brain networks interact with intricate skeletomuscular systems to execute motor behaviors (Carson, 2018). While autistic and non-autistic populations have relatively similar grip strength during childhood, group differences emerge during adolescence and are sustained into adulthood (Abu-Dahab et al., 2013; Alaniz et al., 2015; Travers et al., 2017), thus following a developmental pattern that aligns with observations in other motor domains such as postural control (Minshew et al., 2004). However, it is unclear if the neural mechanisms driving generally decreased grip strength in adolescent and adult autistic populations are present from early childhood or arise concurrently with observable differences in behavior. This information is critical, as it may influence our understanding of foundational brain–body communication in autistic populations and has implications for how we interpret, assess, and implement motor interventions in autistic youth. Therefore, the purpose of this study was to characterize the neural correlates of maximal grip strength in autistic children.

Despite the consistency of motor differences identified in autistic populations, there is a lack of consensus regarding the neurobiological mechanisms by which such differences arise. In adults, diagnosis-dependent differences in the structural and functional neural correlates of grip behavior (Travers et al., 2015; Unruh et al., 2019) as well as broader motor behaviors (Thompson et al., 2017; Lin et al., 2019; Wang et al., 2019; Lepping et al., 2022; McKinney et al., 2022) have previously been identified in the frontal, visual, insular, motor, and cerebellar cortices as well as in the corticospinal tract. However, the results reported across these studies are somewhat inconsistent, leading to the generation of several theories of neuromotor control in the autistic population. Many of these theories center upon differences in sensory processing (Gowen and Hamilton, 2013; Torres et al., 2013; Baum et al., 2015) or cortico-cerebellar communication (Mostofsky et al., 2009; Mosconi et al., 2015). With substantial motor and neural heterogeneity across autistic individuals, it is possible that both theories of motor control are supported by different subsets of the autistic population (such as those with specific co-occurring conditions or cognitive profiles) or may scale with individual differences in specific behavioral, developmental, or contextual features (Insel et al., 2010). Yet, it remains unclear how key sensory and cerebellar networks contribute to fundamental motor behaviors such as the production of grip force or how these theories of neuromotor control apply to pediatric autistic populations, given the developmental nature of autism. One potential reason for this gap in the literature is that there are several challenges with acquiring high-resolution brain images that allow for a more precise examination of brainstem and cerebellar regions, key structures associated with motor behaviors. However, our group has recently optimized diffusion MRI acquisition and processing techniques aimed to mitigate these challenges, thereby allowing us to examine white matter pathways, including brainstem and cerebellar pathways, in children (Guerrero-Gonzalez et al., 2022). Use of these tools to examine white matter microstructure may ultimately lead to more clarity regarding the unique roles that sensory and cortico-cerebellar networks play in not only the production of motor behaviors in autistic children but also the neurobiological basis of broader autism features.

Theoretical models of motor control and empirical evidence from human and non-human primates indicate that sensory and cerebellar networks additionally interact with the lateral grasping network and the corticospinal tract to regulate grip (Doya, 2000; Keisker et al., 2009, 2010; Grafton, 2010; Manto et al., 2012; Schulz et al., 2012; King et al., 2014; Koppelmans et al., 2015; Alahmadi et al., 2017; Borra et al., 2017; Desmurget et al., 2018; Haar and Donchin, 2020; Marneweck and Grafton, 2020). Applying this knowledge to a pediatric sample, our group recently mapped these key white matter tracts in children with no known neurodevelopmental or psychiatric diagnoses (referred to hereafter as “non-autistic”) and identified relationships between grip strength and microstructural coherence of the cortical proprioception input network, lateral grasping network, and corticospinal output pathway but not the cerebellar modification network (Surgent et al., 2023). These findings suggested a potential mechanism of fundamental motor behavior in non-autistic children that is associated with myelination of proprioception and motor-output networks but not cerebellar modification networks. Yet, it remains unclear if these networks similarly contribute to grip strength in autistic children.

Therefore, the first aim of this study was to characterize the microstructural properties of sensorimotor networks as they relate to maximal grip strength in autistic compared to non-autistic children. Based on findings in non-autistic children (Surgent et al., 2023), microstructural properties were examined through free-water eliminated fractional anisotropy (FWE-FA) and quantitative R1 relaxometry, indirect measures concordant with stronger microstructural coherence and increased myelination, respectively. Based on previous reports of relationships between grip behavior and elements of sensorimotor network structure and function in autistic adults (Thompson et al., 2017; Lin et al., 2019; Unruh et al., 2019; Wang et al., 2019; Lepping et al., 2022; McKinney et al., 2022) and previously reported diagnostic group differences in brain-behavior relationships across broader motor domains (Mahajan et al., 2016; Surgent et al., 2021; McKinney et al., 2022), we hypothesized that autistic children would show a distinct pattern of grip-brain relationships compared to non-autistic children, even at this age range when grip strength is behaviorally similar across groups. Specifically, given the various theories of differences in sensory processing (Gowen and Hamilton, 2013; Baum et al., 2015) and cortico-cerebellar communication (Mostofsky et al., 2009; Mosconi et al., 2015) in autistic individuals, we hypothesized that autistic children would differ from non-autistic children in their associations between grip strength and the microstructural properties of the proprioception and/or cerebellar modification networks, as these networks, respectively, underlie sensory input and modification of motor commands.

Given the heterogeneity in autistic motor profiles and the diversity in theories of neuromotor control in autism, a second, exploratory, aim sought to identify additional features that may influence relationships between grip strength and sensorimotor network structure in autistic children. We conducted analyses that tested for the potential of unique grip-microstructure relationships based on factors previously found to be associated with motor behavior in autistic populations including autism features (MacDonald et al., 2013), attention-deficit/hyperactivity disorder (ADHD) features (Mattard-Labrecque et al., 2013; Mahajan et al., 2016), sensory features (Surgent et al., 2021), and variation in IQ (Travers et al., 2018; Surgent et al., 2021). In addition to being associated with motor behavior in autistic youth and being commonly associated with behavioral profiles of autistic individuals, each of these factors has also been associated with brain variation in autistic populations (Mahajan et al., 2016; Bedford et al., 2020; Surgent et al., 2021, 2022; McKinney et al., 2022). Together, this suggests that individual differences in autism features, ADHD features, sensory features, and IQ may not only exist concurrently with individual differences in motor behavior but also may have unique neural signatures that influence the brain mechanisms of motor control in autistic youth. Therefore, we hypothesized that within the autistic group, grip strength-white matter relationships would be moderated by one or more of these factors. Through examination of these potential moderating effects, we aimed to disentangle some of the heterogeneity in brain-behavior relationships within autistic youth with the ultimate goal of identifying how subsets of the autistic population may align with or diverge from the established theories of neuromotor control in autism.

## 2 Methods

### 2.1 Participants

Participants included 68 autistic children and 70 non-autistic children, between the ages of 6.0 and 11.0 years old. All participants were required to communicate verbally and have an IQ score greater than 60 using the Wechsler Abbreviated Scale of Intelligence, 2nd Edition (WASI-2) (Wechsler and Hsiao-pin, 2011) or the Kaufman Brief Intelligence Test-Second Edition (KBIT-2) (Kaufman and Kaufman, 2004). None of the participants had a previous diagnosis of tuberous sclerosis, Down syndrome, fragile X, hypoxia-ischemia, notable and uncorrected hearing or vision loss, or a history of severe head injury. The institutional review board at the University of Wisconsin-Madison approved all procedures. In each case, the child participant provided assent and a parent or guardian provided informed consent.

To confirm previous community diagnoses of autism spectrum disorder (ASD), participants in the autistic group were comprehensively evaluated for ASD by meeting cutoffs on either (1) the Autism Diagnostic Observation Schedule, 2nd edition (ADOS-2; cutoff = 8) (Lord et al., 2012) or the Autism Diagnostic Interview-Revised (ADI-R) (Lord et al., 1994). Six participants narrowly missed cutoff on the ADOS-2, however they were included in the autistic group after a record review with a licensed clinical psychologist. All six met cutoff on both the Social Responsiveness Scale, second edition (SRS-2; cutoff = 60) (Constantino and Gruber, 2012) and the Social Communication Questionnaire (SCQ; cutoff = 15) (Rutter et al., 2003).

Non-autistic participants were required to score less than 8 on the SCQ (Rutter et al., 2003). Participants were excluded from the non-autistic group if they had a previous diagnosis of any neurodevelopmental disorder including ADHD, bipolar disorder, major depressive disorder, or if they had a first-degree relative with ASD. Specific demographic information about the participant sample can be found in Table 1.

**Table 1:** Demographic information for participant sample.

	Non-autistic	Autistic	*t*-value	*p*
	*n* = 70	*n* = 68		
Age (years), Mean (SD)	8.34 (1.36)	8.40 (1.36)	−0.3	0.80
Sex, % Female	34%	19%	3.5^^^	0.06
Average head motion (AVD), Mean (SD)	0.53 (0.42)	0.67 (0.49)	−1.7	0.10
Hand preference, % Right-handed	85.7%	79.4%	1.6^^^	0.19
Maximum grip, kg (bilateral average), Mean (SD)	13.35 (3.75)	12.85 (4.34)	0.8	0.43
Maximum grip, kg (right hand), Mean (SD)	13.74 (3.94)	13.18 (4.51)	0.8	0.39
Maximum Grip, kg (left hand), Mean (SD)	12.96 (3.77)	12.52 (4.37)	0.7	0.50
IQ, Mean (SD)	114.41 (12.14)	106.64 (19.11)	2.8	0.01
SRS total score, Mean (SD)	21.94 (13.55)	97.13 (26.84)	−20.8	<0.001
SCQ total score, Mean (SD)	1.45 (1.88)	19.71 (6.46)	−22.5	<0.001
SEQ total score, Mean (SD)	138.5 (21.3)	225.4 (43.1)	−15.0	<0.001
RBS-R total score, Mean (SD)	2.43 (3.67)	32.69 (22.98)	−10.8	<0.001
NICHQ-VAS total score, Mean (SD)	9.4 (6.0)	29.3 (11.3)	−13.5	<0.001
ADHD status	0%	60%	22.7^^^	<0.001
Taking centrally activating medication, %	1%	36%	22.5^^^	<0.001

^Indicates *χ*^2^ value instead of *t*-value. ADHD status determined by meeting cut off on the NICHQ-VAS inattention or hyperactivity subscales.AVD, Average Volume Displacement (Bastiani et al., 2019), SRS, Social Responsiveness Scale (Constantino and Gruber, 2012), SCQ, Social Communication Questionnaire (Rutter et al., 2003), RBS-R, Repetitive Behavior Scale-Revised (Bodfish et al., 2000), NICHQ-VAS, National Initiative for Children’s Healthcare Quality Vanderbilt Assessment Scales (American Academy of Pediatrics and National Institute for Children’s Health Quality, 2002), SEQ, Sensory Experience Questionnaire (Baranek et al., 2006).

### 2.2 Grip strength assessment

Maximal grip strength was measured using a Jamar hand dynamometer (Heaton et al., 1991). Each participant stood with their arm at their side (bent at a 90-degree angle) and squeezed the dynamometer with one hand as hard as possible without moving the rest of their body. Grip strength, measured in kilograms, was recorded from both hands across 10 trials (5 trials per hand). The average of the maximum grip strength from the right- and left-hand trials was used in the analysis.

### 2.3 Additional behavior assessments

Parent reported measures were collected to assess ADHD features (National Initiative for Children’s Healthcare Quality Vanderbilt Assessment Scales [NICHQ-VAS]) (American Academy of Pediatrics and National Institute for Children’s Health Quality, 2002), sensory features (Sensory Experience Questionnaire [SEQ-3]) (Baranek et al., 2006), and autism features (SRS-2, SCQ, and Repetitive Behavior Scale-Revised [RBS-R]) (Bodfish et al., 2000) in all participants. On all assessments, higher scores are associated with more prominent features. Further information about these assessments can be found in Supplementary material.

### 2.4 MRI data acquisition and processing

Two types of structural magnetic resonance imaging (MRI) data (diffusion and T1-weighted) were acquired on a 3 T GE Discovery MR750 scanner (Waukesha, WI) using a 32-channel phased array head coil (Nova Medical, Wilmington, MA). Our collection protocols for these data represent state-of-the art MRI acquisitions that aim to limit distortions and produce biologically plausible representations of the pediatric brain *in vivo*. Specifically, diffusion-weighted images (DWIs) were obtained using a multi-shell spin-echo echo-planar imaging (EPI) pulse sequence (9 directions at b = 350 s/mm^2^, 18 directions at b = 800 s/mm^2^, and 36 directions at b = 2000 s/mm^2^, and 6 non-diffusion-weighted [b = 0 s/mm^2^] volumes; Repetition Time (TR)/Time to Echo (TE) = 9000/74.4 ms; Field of View (FOV) = 230 mm × 230 mm, in-plane resolution 2.4 mm × 2.4 mm, interpolated to 1.8 mm × 1.8 mm; 76 overlapping slices 3.6 mm thick with slice centers spaced every 1.8 mm). An additional six, non-diffusion-weighted volumes with the reverse phase-encoded direction were collected for use in correcting susceptibility-induced artifacts (Andersson et al., 2003). 3D T1-weighted (T1w) images were obtained using an MPnRAGE sequence with 1mm^3^ isotropic resolution. The MPnRAGE pulse sequence combines magnetization preparation using inversion recovery with a rapid 3D radial *k*-space readout (Kecskemeti et al., 2016, 2018). It also employs a retrospective head-motion correction, allowing for highly repeatable tissue-specific segmentation and quantitative T1 (qT1) mapping (Kecskemeti et al., 2018, 2021; Kecskemeti and Alexander, 2020a,b) even with large amounts of head motion.

DWIs were preprocessed to reduce noise (Veraart et al., 2016a,b), Gibbs ringing (Kellner et al., 2016), and artifacts caused by motion, eddy current (Andersson et al., 2016, 2017; Andersson and Sotiropoulos, 2016) and EPI distortions (Andersson et al., 2003) and subsequently processed in accordance to the TiDi-Fused workflow (Guerrero-Gonzalez et al., 2022), to enhance the apparent spatial resolution. Briefly, as part of the TiDi-Fused workflow, transformations used to map the DWI b = 0 volume to the MPnRAGE T1-weighted images were computed using a rigid-body boundary-based registration (BBR) (Greve and Fischl, 2009) routine in the FreeSurfer image analysis suite (Dale et al., 1999). The entire DWI series was then transformed using ANTs (Avants et al., 2011) with cubic B-spline interpolation up-sampled to the T1w resolution (1 mm isotropic). The rotational component of the rigid body transformation was then applied to the DWI encoding directions. The enhanced apparent resolution provided by this workflow allows for more accurate delineation of white matter pathways (Guerrero-Gonzalez et al., 2022). Additionally, the average relative voxel displacement between volumes acquired during the DWI scan was estimated using *eddy_qc* and utilized to quantify participant head motion (Andersson and Sotiropoulos, 2016).

Free water elimination (FWE) diffusion tensor imaging (DTI) was used to estimate diffusion tensors. The FWE approach has been shown to produce more complete, anatomically plausible tract representations in regions with suspected CSF partial volume artifacts (Hoy et al., 2015) and therefore was used to generate more accurate tensor-based estimates in cortical and subcortical tracts of interest. FWE fractional anisotropy (FWE-FA) maps were generated from the FWE tensor maps (Fick et al., 2019). Quantitative T1 maps of the brain were obtained from MPnRAGE images from which R1 estimates (R1 = 1/qT1) were calculated at each voxel (Kecskemeti and Alexander, 2020a). All structural maps passed a visual inspection for processing artifacts prior to statistical analyses.

### 2.5 White matter tract definition

#### 2.5.1 White matter tractography

**Figure 1:**
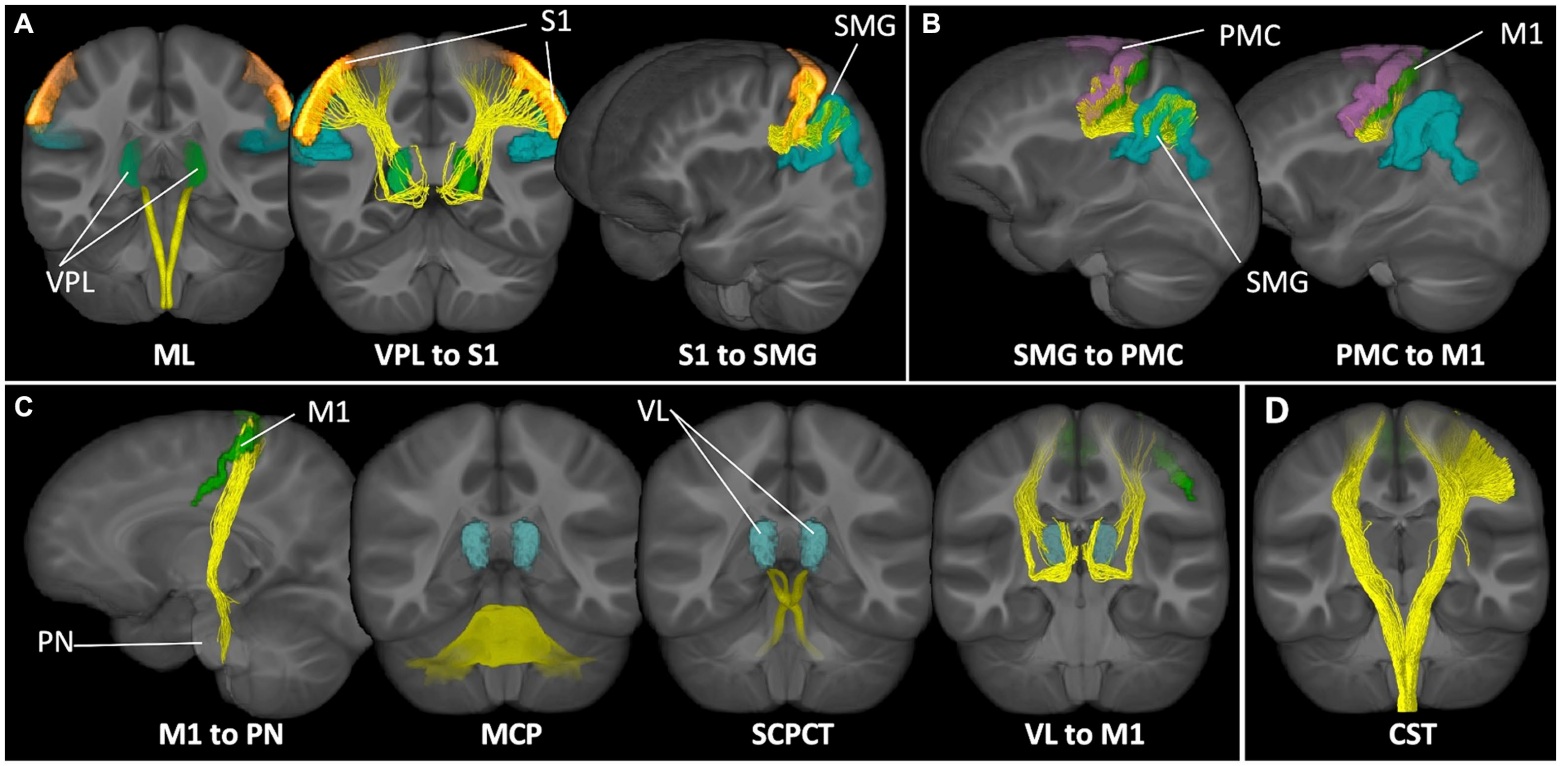
Sensorimotor networks of interest. Tract representations of pathways included in the **(A)** proprioception input network, **(B)** lateral grasping network, **(C)** cerebellar modification network, and **(D)** corticospinal tract. ML, medial lemniscus; VPL, ventral posterolateral nucleus of the thalamus; S1, primary somatosensory cortex; SMG, supramarginal gyrus; PMC, premotor cortex; M1, motor cortex; PN, pontine nuclei; MCP, middle cerebellar peduncle; SCPCT, superior cerebellar peduncle, cerebello-thalamic tract; VL, ventrolateral nucleus of the thalamus; CST, corticospinal tract.

As described in (Surgent et al., 2023), whole brain tractograms were constructed MRtrix3 white matter tracking algorithms (Tournier et al., 2019). Anatomically constrained tractography (Smith et al., 2012) was performed based on multi-shell multi-tissue constrained-spherical deconvolution (msmt-CSD) (Jeurissen et al., 2014; Dhollander et al., 2016) and probabilistic tracking (iFOD2) (step size: 0.5 mm; maximum length: 250 mm; minimum length: 10 mm; 20,000,000 streamlines) (Tournier et al., 2010). Tracts were seeded from a whole brain white matter mask and tracking was terminated if a streamline exited the mask. Following spherical-deconvolution informed filtering of the tractograms (SIFT2) (Smith et al., 2015), connectomes representing white matter pathways spanning gray matter regions of interest were constructed using parcellations derived from FreeSurfer automatic segmentation (Fischl et al., 2008; Destrieux et al., 2010; Iglesias et al., 2018). See Supplementary Table S1 for details about tract inclusion and exclusion criteria for defining intrahemispheric white matter pathways. See Figure 1 for representations of the tracts spanning each network of interest. Tracts were visually inspected to verify integrity and biological validity. All participants included in the sample had biologically plausible tract reconstructions. The weighted median of each tract bundle was then calculated and used in analyses.

#### 2.5.2 Brainstem white matter atlas

To increase biological accuracy and avoid common challenges with white matter fiber tracking in relatively small and highly intricate brainstem tracts (Tang et al., 2018), brainstem-based white matter tracts (medial lemniscus [ML], middle cerebellar peduncle [MCP], superior cerebellar peduncle tracts to the thalamus [SCPCT]) were defined using a probabilistic brainstem connectome atlas (Tang et al., 2018). As in previous work (Surgent et al., 2022, 2023), affine and diffeomorphic transformations (Avants et al., 2011) were used to map tracts to a T1w study-specific template that was aligned with the MNI152 T1w image. Bundles were warped to the native subject space, with linear interpolation, using the transforms generated during population template estimation. Summary diffusion measures of the ML, MCP, and SCPCT tracts were calculated using the weighted median based on probabilistic tractography visitation counts (normalized to values between 0 and 1 at each voxel). All bundles were quality assessed by outlier analysis of the summary measures.

### 2.6 Statistical analysis

To determine the extent to which relationships between grip and microstructure were similar or different in autistic compared to non-autistic children, global general linear models were constructed predicting FWE-FA or R1 from maximal grip strength, diagnostic group (autistic vs. non-autistic), tract (10 tracts total [three proprioception input network, two lateral grasping network, four cerebellar modification network, one corticospinal output pathway]), and their two- and three-way interactions, while accounting for age and sex and including a random effect for participant. *Post-hoc*, tract-specific analyses were conducted to examine grip strength-by-group interaction effects as well as grip strength-by-tract interaction effects. All *post-hoc* analyses were corrected for multiple comparisons using false discovery rate (FDR corrected, *p* < 0.05) (Benjamini and Hochberg, 1995). All FWE-FA models additionally controlled for average head motion during the DWI scan.

To assess sensorimotor tract model fit, we sought to determine if within-group brain-behavior heterogeneity was consistent across the autistic and non-autistic samples. Results of within-tract post-hoc analyses in the autistic group were compared to parallel analyses in a non-autistic group (previously described in Surgent et al., 2023) using Fisher’s *F*-tests. This provided information regarding statistically significant differences in the variance explained by models predicting structural properties of each sensorimotor tract from grip strength and relevant covariates in autistic compared to non-autistic groups.

To determine if higher variances in the brain-behavior relationships within the autistic group were driven by additional factors that may influence the relationship between grip strength and sensorimotor white matter, exploratory analyses were conducted using moderated global mixed-effects linear regression modeling. Moderators of interest included continuous measures of autism features, sensory features, ADHD features, and IQ. Significant three-way interaction effects were subsequently examined with post-hoc analyses to determine the extent to which these factors moderated grip-white matter relationships on a tract-by-tract basis. While analyses examined ADHD features continuously as a total score, additional demographic information about the autistic participants with elevated or reduced ADHD features (based on meeting cutoff criteria on the inattention or hyperactivity domain of the NICHQ-VAS) can be found in Supplementary Table S2.

## 3 Results

### 3.1 Comparison of sensorimotor-grip relationships in autistic and non-autistic children

**Figure 2:**
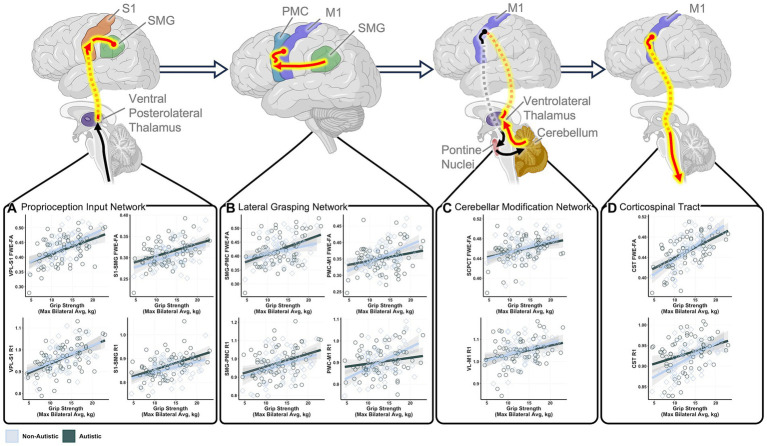
Statistically significant relationships between grip strength and sensorimotor tract structure in autistic and non-autistic children. Schematic tract representations with tracts that demonstrated significant grip strength-white matter structural relationships displayed in red. Scatter plots show significant correlations between maximum grip strength and structural measures of the **(A)** cortical proprioception input network tracts, **(B)** lateral grasping network tracts, **(C)** cerebellar modification network tracts, and **(D)** corticospinal tract. All relationships control for age and sex. FWE-FA relationships additionally control for head motion during the DWI scan. All tests are corrected for multiple comparisons using FDR *p* < 0.05. Network schematics created with BioRender.com.

Tracts for comparison can be seen in Figure 1. Global modeling revealed no significant main effect of diagnostic group (autistic vs. non-autistic) or interaction effects between grip strength and diagnostic group predicting FWE-FA (Table 2). A significant three-way interaction effect of grip strength-by-diagnostic group-by-tract was found to predict R1 (Table 2), however within-tract post-hoc analyses did not reveal any significant grip strength-by-diagnostic group interaction effects after FDR correction (Supplementary Table S3). *Post-hoc* analyses of sensorimotor-grip relationships across all participants at the individual tract level revealed significant positive relationships between grip strength and cortical proprioception input network tract FWE-FA and R1 (Figure 2A), lateral grasping network FWE-FA and R1 (Figure 2B), cerebellar modification network SCPCT FWE-FA and VL-M1 R1 (Figure 2C), and corticospinal tract FWE-FA and R1 (Figure 2D; Table 3).

**Table 2:** Results of linear mixed effect models predicting sensorimotor network microstructure from grip strength in autistic vs. non-autistic children.

Metric of interest	Predictor	Sum of squares	Mean square	*F*	*p*
*FWE-FA*					
	Grip strength	0.0306	0.0306	33.43	<0.001
	Diagnostic group	0.0029	0.0029	3.21	0.08
	Sensorimotor tract	8.3218	0.9246	1011.64	<0.001
	Age	0.0044	0.0044	4.84	0.03
	Sex	0.0022	0.0022	2.44	0.12
	Head motion	0.0450	0.0450	49.27	<0.001
	Grip strength × Diagnostic group	0.0012	0.0012	1.29	0.26
	Grip strength × Tract	0.0322	0.0036	3.91	<0.001
	Diagnostic group × Tract	0.0420	0.0047	5.11	<0.001
	Grip strength × Diagnostic group × Tract	0.0092	0.0010	1.12	0.35
*R1*					
	Grip strength	0.0243	0.0243	15.90	<0.001
	Diagnostic group	0.0009	0.0009	0.61	0.44
	Sensorimotor tract	3.5373	0.3930	257.04	<0.001
	Age	0.0045	0.0045	2.93	0.09
	Sex	0.0008	0.0008	0.53	0.47
	Grip strength × Diagnostic group	0.0012	0.0012	0.76	0.39
	Grip strength × Tract	0.1100	0.0122	7.99	<0.001
	Diagnostic group × Tract	0.0213	0.0024	1.55	0.13
	Grip strength × Diagnostic group × Tract	0.0285	0.0032	2.07	0.03

**Table 3:** Results of within-tracts *post hoc* analyses predicting structural properties from maximal grip strength across autistic and non-autistic children.

Metric of interest	Tract of interest	*b*	*SE*	95% Confidence interval		*t*	*p* (FDR-adjusted)
				Lower	Upper		
**FWE-FA**							
*Proprioception input network*							
	ML	0.001	0.001	−0.0003	0.0024	1.48	0.17
	VPL Thal-S1	0.005	0.001	0.0030	0.0068	5.13	<0.001
	S1-SMG	0.002	0.001	0.0011	0.0037	3.75	<0.001
*Lateral grasping network*							
	SMG-PMC	0.004	0.001	0.0017	0.0061	3.48	0.001
	PMC-M1	0.004	0.001	0.0016	0.0055	3.60	0.001
*Cerebellar modification network*							
	M1-PN	0.001	0.001	−0.0006	0.0022	1.17	0.25
	MCP	0.001	0.001	−0.0004	0.0026	1.45	0.17
	SCPCT	0.001	0.001	0.0002	0.0025	2.29	0.04
	VL Thal -M1	0.002	0.001	−0.0006	0.0040	1.47	0.17
*Corticospinal output*							
	CST	0.004	0.001	0.0023	0.0047	5.67	<0.001
**R1**							
*Proprioception input network*							
	ML	0.000	0.001	−0.0011	0.0015	0.30	0.94
	VPL Thal-S1	0.006	0.001	0.0035	0.0091	4.46	0.001
	S1-SMG	0.004	0.001	0.0018	0.0061	3.61	0.001
*Lateral grasping network*							
	SMG-PMC	0.007	0.002	0.0035	0.0111	3.79	0.001
	PMC-M1	0.006	0.002	0.0024	0.0092	3.40	0.002
*Cerebellar modification network*							
	M1-PN	0.001	0.001	−0.0012	0.0032	0.91	0.52
	MCP	0.000	0.001	−0.0015	0.0016	0.07	0.94
	SCPCT	0.000	0.001	−0.0013	0.0015	0.14	0.94
	VL Thal-M1	0.003	0.001	0.0007	0.0062	2.51	0.02
*Corticospinal output*							
	CST	0.003	0.001	0.0013	0.0050	3.36	0.002

All post hoc analyses controlled for age, and sex.FWE-FA analyses additionally controlled for head motion during the DWI scan.

While at the group level, grip strength did not significantly differ in autistic compared to non-autistic children (Table 1), additional analyses were conducted to test if the heterogeneity in grip strength predictions of sensorimotor white matter within the autistic group was similar to the heterogeneity in grip strength predictions of sensorimotor white matter within the non-autistic group. Results of these model comparisons revealed that, generally, grip strength was a stronger predictor of sensorimotor tract structural properties within non-autistic children as indexed by higher beta values and smaller residual standard error (Supplementary Figure S1). Statistical comparisons of variance showed that this was especially the case for FWE-FA and R1 in cerebellar modification network tracts and the PMC-M1 tract of the lateral grasping network, all of which showed statistically higher variance within the autistic group (Supplementary Table S4).

### 3.2 Dimensional analysis of moderating factors for the relationship between grip strength and sensorimotor white matter

**Figure 3:**
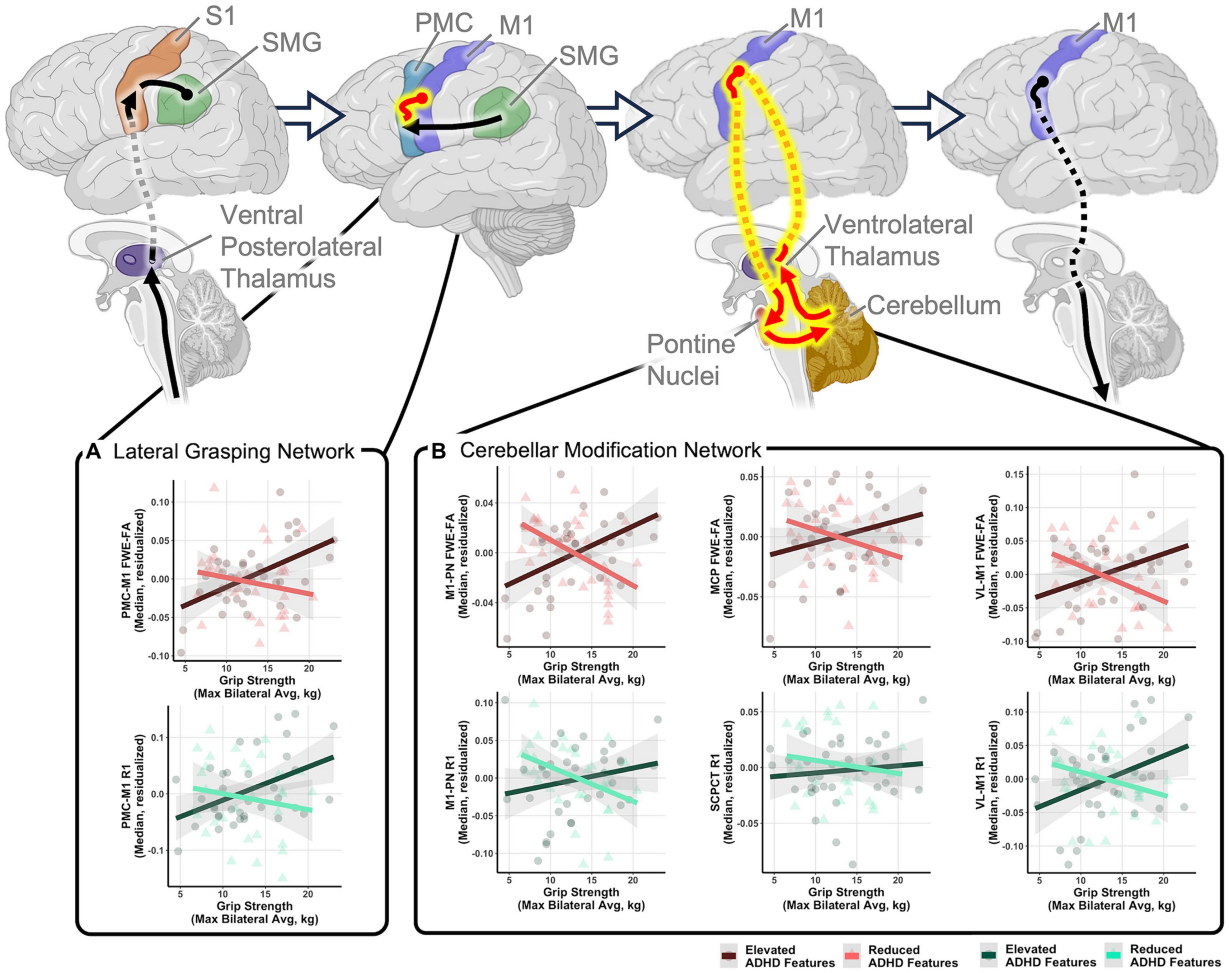
Relationships between grip strength and sensorimotor tract structure in autistic children moderated by ADHD features. Schematic tract representations with tracts displayed in red representing those that with significant grip strength-white matter structural relationships that were moderated by ADHD features. Scatter plots show relationships between maximum grip strength and structural measures of the **(A)** lateral grasping network and **(B)** cerebellar modification network tracts. Relationships are shown in autistic children with elevated ADHD features (ADHD feature scores greater than the group mean; dark red and dark green) and children with reduced ADHD features (ADHD feature scores less than the group mean; light red and light green) to illustrate moderating effects of ADHD features but moderation analyses were performed using a dimensional characterization of ADHD features. All relationships control for age and sex. FWE-FA relationships additionally control for head motion during the DWI scan. All tests are corrected for multiple comparisons using FDR *p* < 0.05. Network schematics created with BioRender.com.

Exploratory analyses were conducted to determine if specific behavioral factors may influence the relationship between grip strength and sensorimotor tract structure. Global general linear models assessed the moderating power of four behavioral factors (ADHD features, autism features, sensory features, IQ) on the relationships between grip strength and sensorimotor tract structural properties within the autistic group. Results indicated that individual differences in ADHD features significantly moderated the relationship between grip strength and tract structural features as indexed by significant grip strength-by-ADHD feature-by-tract interaction effects predicting both FWE-FA and R1 (Table 4). *Post-hoc* analyses revealed that ADHD features specifically moderated sensorimotor-grip relationships in the cerebellar modification network and PMC-M1 tracts (Table 5). The moderating effect of ADHD features on the grip strength-sensorimotor network relationships in autistic children is depicted in Figure 3. No other factors tested were found to be significant moderators (Supplementary Tables S5–S8). Follow-up analyses found no significant differences in grip strength in autistic children with elevated ADHD features compared to those with reduced ADHD features (Supplementary Table S2). Further follow-up analyses tested if these observed grip strength-by-ADHD feature relationships with white matter were driven by medication status. No significant relationships were found between grip strength, medication status, nor sensorimotor white matter structure (Supplementary Table S9).

**Table 4:** Results of linear mixed effect models predicting sensorimotor network microstructure from ADHD features and maximum grip strength in autistic children.

Metric of interest	Predictor	Sum of squares	Mean square	*F*	*p*
*FWE-FA*					
	Grip strength	0.004	0.004	3.69	0.06
	ADHD features	0.000	0.000	0.23	0.64
	Sensorimotor tract	0.434	0.048	48.53	<0.001
	Age	0.008	0.008	7.76	0.007
	Sex	0.001	0.001	0.70	0.41
	Head motion	0.021	0.021	21.16	<0.001
	Grip strength × ADHD features	0.011	0.011	11.38	0.001
	Grip strength × Tract	0.028	0.003	3.13	0.001
	ADHD features × Tract	0.011	0.001	1.21	0.29
	Grip strength × ADHD features × Tract	0.028	0.003	3.13	<0.001
*R1*					
	Grip strength	0.003	0.003	1.96	0.17
	ADHD features	0.001	0.001	0.33	<0.57
	Sensorimotor tract	0.211	0.023	14.66	0.001
	Age	0.006	0.006	3.82	0.06
	Sex	0.000	0.000	0.20	0.66
	Grip strength × ADHD features	0.009	0.009	5.67	0.02
	Grip strength × Tract	0.047	0.005	3.28	0.001
	ADHD features × Tract	0.011	0.001	0.78	0.64
	Grip strength × ADHD features × Tract	0.044	0.005	3.06	0.001

**Table 5:** Results of within-tracts *post hoc* analyses predicting structural properties from maximal grip strength-by-ADHD features in autistic children.

Metric of interest	Tract of interest	*b*	*SE*	95% Confidence interval		*t*	*p* (FDR-adjusted)
				Lower	Upper		
**FWE-FA**							
*Proprioception input network*							
	ML	0.0000	0.0001	−0.0001	0.0001	0.15	0.88
	VPL Thal-S1	0.0002	0.0001	0.0000	0.0003	1.59	0.19
	S1-SMG	0.0001	0.0001	−0.0001	0.0002	1.17	0.35
*Lateral grasping network*							
	SMG-PMC	0.0002	0.0001	0.0000	0.0004	2.08	0.08
	PMC-M1	0.0003	0.0001	0.0001	0.0005	3.25	0.01
*Cerebellar modification network*							
	M1-PN	0.0003	0.0001	0.0001	0.0004	3.86	0.001
	MCP	0.0002	0.0001	0.0000	0.0003	2.37	0.04
	SCPCT	0.0001	0.0001	−0.0001	0.0001	0.40	0.85
	VL Thal-M1	0.0003	0.0001	0.0001	0.0006	2.51	0.04
*Corticospinal output*							
	CST	0.0000	0.0001	−0.0001	0.0001	−0.30	0.85
**R1**							
*Proprioception input network*							
	ML	0.0000	0.0001	−0.0001	0.0002	0.29	0.84
	VPL Thal-S1	0.0002	0.0001	−0.0001	0.0005	1.49	0.28
	S1-SMG	0.0001	0.0001	−0.0002	0.0003	0.52	0.84
*Lateral grasping network*							
	SMG-PMC	0.0002	0.0002	−0.0002	0.0005	1.01	0.53
	PMC-M1	0.0004	0.0002	0.0001	0.0007	2.55	0.04
*Cerebellar modification network*							
	M1-PN	0.0004	0.0001	0.0001	0.0006	3.16	0.02
	MCP	0.0000	0.0001	−0.0002	0.0002	0.26	0.84
	SCPCT	0.0002	0.0001	0.0000	0.0003	2.13	0.04
	VL Thal-M1	0.0004	0.0001	0.0001	0.0007	3.08	0.02
*Corticospinal output*							
	CST	0.0000	0.0001	−0.0002	0.0002	−0.20	0.84

All *post-hoc* analyses controlled for age, and sex.FWE-FA analyses additionally controlled for head motion during the DWI scan.

## 4 Discussion

To characterize the structural neural underpinnings of grip strength in autistic children, we assessed the relationships between maximal grip strength and microstructural features of foundational sensorimotor networks. We found that grip strength was associated with structural aspects of the proprioception, lateral grasping, and corticospinal networks in autistic children in a way that was generally similar to non-autistic children. This suggests that at the diagnostic-group level, foundational neural contributions to motor behavior are similar among autistic and non-autistic individuals during childhood, a time when grip strength is also similar between the groups. However, when we assessed the autistic group in the context of additional behavioral features that may influence relationships between motor behavior and white matter microstructure, unique relationships emerged. Specifically, ADHD features were found to moderate the relationships between grip strength and microstructural aspects of lateral grasping and cerebellar-based networks. These differing neural correlates provide evidence of distinct contributions of cortical and cerebellar networks in autistic children that may scale with the prominence of ADHD features. Further, these findings help to in part explain the heterogeneity in autistic brain-behavior relationships and give a more nuanced understanding of the mechanisms underlying neuromotor control in autistic children. This work has implications for the understanding of motor differences in autistic populations and is discussed in more detail.

At the group level, autistic and non-autistic children had similar grip strength and similar relationships between grip strength and microstructural coherence of the lateral grasping, the cortical proprioception input (VPL-S1, S1-SMG), and aspects of the cerebellar modification (SCPCT and VL-M1) networks as well as the corticospinal tract. These behavioral findings corroborate past work showing that grip strength is similar in autistic and non-autistic groups during childhood with diagnostic group differences emerging during adolescence (Travers et al., 2017). Further, brain-behavior relationships support theories of neuromotor control that emphasize foundational nature of the SMG, PMC, S1, and M1/corticospinal tract (Haar and Donchin, 2020). Importantly, our results demonstrate the translatability of these theories of neuromotor control to a neurodiverse pediatric population. Further, myelination, approximated via measures of R1, may be the neurobiological mechanism by which these tracts contribute to grip strength. Interestingly, the developmental timeline of myelination group differences in autistic and non-autistic youth (Deoni et al., 2015) approximates that of grip strength group differences (Travers et al., 2017), perhaps suggesting that myelin development in these brain regions underlies the communication efficiency of foundational brain networks and contributes to the emergence of population-level grip strength differences in adulthood. However, it is important to note that R1 is also sensitive to concentrated iron in regions of high neuroinflammation (Pontillo et al., 2022), a condition previously reported in autistic individuals (van Tilborg et al., 2018; Han et al., 2021). Given the restricted age range of the present sample, we were unable to deeply characterize the effects of age on these brain-behaviors relationships. Therefore, future work employing longitudinal based designs that characterize grip-white matter relationships in the times before, during, and after behavioral group-differences in grip strength emerge will be needed to precisely determine how closely these developmental trajectories align.

While generally similar grip-microstructure relationships were found across non-autistic and autistic children, considerable heterogeneity was found within the PMC-M1 and cerebellar modification network tracts in the autistic group, suggesting that the extent to which these networks are relied upon is not consistent across all autistic children. Subsequent analyses suggested that ADHD features moderate relationships between grip strength and structural properties of the PMC-M1 and cerebellar modification network tracts in autistic children. These relationships provide evidence for both sensory and cerebellar-based theories of autistic neuromotor control that are dependent upon the extent to which ADHD features are present. Specifically, grip-microstructure associations in autistic children with reduced ADHD features suggest that cerebellar feedback hinders the production of strong grip force, potentially via less reliable cerebellar-based error correction of cortical motor commands (Mosconi et al., 2015) and thus align with past work supporting cerebellar-based theories of neuromotor control in autism (Unruh et al., 2019; Wang et al., 2019; McKinney et al., 2022). Conversely, microstructure-grip findings in autistic children with elevated ADHD features suggest a need for increased cerebellar based signaling to detect and correct sensorimotor integration errors, potentially supporting theories of altered sensory integration in subgroups of autistic children (Gowen and Hamilton, 2013; Haar and Donchin, 2020) and aligning with past work supporting sensory-based theories of neuromotor control in autism. Together, our findings suggest that altered cerebellar signaling may differently contribute to motor behaviors in autistic children depending upon how consistent each child’s behavioral profile is with ADHD features. Importantly, future work must seek to investigate the dynamic relationships between ADHD features, brain structure, and grip strength over time to determine if subgroups of autistic individuals with and without ADHD show distinct developmental trajectories of grip strength or unique trajectories of brain-behavior relationships.

Further, the moderating effect of ADHD within the autism group suggests alternative underlying mechanisms of motor control in some autistic children, even when the resulting behavior (i.e., grip strength) is similar. Therefore, simply observing behavior without the additional context of neurodivergent features or neuroimaging measures, may not provide sufficient information to predict underlying neural correlates. It then follows that therapeutic approaches that assume a particular pattern of neural reliance may be less effective in some populations of autistic children compared to others, as evidenced by the diversity in motor intervention efficacies reported in autistic populations (Ruggeri et al., 2020). Therefore, future work may need to take a more granular approach to autism intervention by accounting for co-occurring conditions to improve pediatric motor outcomes. Moreover, future research should continue to examine the impact of co-occurring conditions on brain-behavior relationships within the autism spectrum.

These results should be interpreted with consideration of the study limitations. Importantly, our study was limited to children who communicated verbally and were able to navigate the sensorimotor demands of the MRI environment. Therefore, our findings cannot generalize to broader autistic populations who may have different neural correlates of motor behavior from those presented here. Our study also assessed microstructural neural correlates of grip strength cross-sectionally and *in vivo* making it impossible to identify causal patterns in grip-brain relationships or the precise cellular mechanisms driving these findings. Future work employing longitudinal grip strength assessment in humans and histological validation in non-human models may help to precisely identify the causal mechanisms and underlying cellular catalysts of these relationships. Finally, we did not include a sample of non-autistic children with ADHD, thus leaving the question of how networks contribute to behavior in non-autistic children with ADHD unanswered. Additionally, ADHD features were assessed via caregiver-reported measures and not through a full clinical evaluation. Future work may employ similar methodologies with larger cohorts of autistic and non-autistic children with clinically diagnosed ADHD to validate these findings and determine the extent to which brain-behavior relationships are distinct in these groups.

In all, we found that when looking broadly across autistic and non-autistic children, similar relationships between sensorimotor networks and grip strength exist, emphasizing the foundational nature of these networks for motor behavior across neurodiverse pediatric populations. However, when brain-behavior heterogeneity was considered in the autistic group, a different pattern emerged. We found that individual differences in grip-microstructure relationships of cortical and cerebellar tracts were dependent upon ADHD features, suggesting that the prominence of ADHD features in autistic children may be indicative of how reliant the brain is on cerebellar or sensory networks for the execution of fundamental motor tasks. Together, these results provide critical information about the biological basis of motor behavior in autistic children and demonstrate the importance of considering co-occurring conditions when evaluating brain-behavior relationships in heterogeneous autistic populations.

## Data availability statement

The datasets presented in this study can be found in online repositories. The names of the repository/repositories and accession number(s) can be found below: A portion of these data are openly available in National Institute of Mental Health Data Archive at http://doi.org/10.15154/1523353, reference number 3088. The remaining data that support the findings of this study are available from the corresponding author, upon reasonable request.

## Ethics statement

The studies involving humans were approved by University of Wisconsin-Madison Health Sciences Institutional Review Board. The studies were conducted in accordance with the local legislation and institutional requirements. Written informed consent for participation in this study was provided by the participants’ legal guardians/next of kin.

## Author contributions

OS: Conceptualization, Data curation, Formal analysis, Methodology, Visualization, Writing – original draft, Writing – review & editing. JG-G: Methodology, Software, Writing – review & editing. DD: Methodology, Software, Writing – review & editing. NA: Methodology, Software, Writing – review & editing. GK: Methodology, Writing – review & editing. SK: Software, Writing – review & editing. AA: Methodology, Resources, Supervision, Writing – review & editing. JL: Funding acquisition, Resources, Writing – review & editing. BT: Conceptualization, Funding acquisition, Investigation, Methodology, Resources, Supervision, Writing – review & editing.
