# Supplementary material for: Microstructural neural correlates of maximal grip strength in autistic children: the role of the cortico-cerebellar network and attention-deficit/hyperactivity disorder features
Source: Front Integr Neurosci. 2024 May 14;18:1359099. doi: 10.3389/fnint.2024.1359099 (PMC11130426; doi:10.3389/fnint.2024.1359099)
Supplement: Supplementary file 1 [file Data_Sheet_1.PDF]

Supplementary Methods.

1. Behavioral measures used in exploratory moderation analyses.

1.1. National Initiative for Children's Healthcare Quality Vanderbilt Assessment Scales (NICHQ-VAS). The NICHQ-VAS is a caregiver reported measure that was used to assess ADHD features. The NICHQ-VAS assesses both features of predominantly inattentive and predominantly hyperactive/impulsive subtypes of ADHD through two components: symptom assessment and impairment in performance (American Academy of Pediatrics & National Institute for Children's Health Quality, 2002). The total score symptom assessment score was used in analyses to quantify prominence of ADHD features in each participant. Total scores were examined rather than subscale (hyperactive/impulsive or initiative) scores as subscales each showed low variance when assessed independently.

1.2. Sensory Experience Questionnaire (SEQ-3.0). The Sensory Experiences Questionnaire 3.0 (SEQ-3.0) is a parent report measure used to characterize sensory features in children 2-12 years old. The assessment has been validated for use in autistic and non-autistic children (Baranek, 2009). This questionnaire contains 97 quantitative items that measure the frequency of sensory-related behaviors across four key patterns (hyporesponsiveness, hyperresponsiveness, repetitions and seeking behaviors, and enhanced perception) and all sensory modalities (auditory, visual, tactile, gustatory/olfactory, and vestibular/proprioceptive; (Ausderau et al., 2016)). All items utilize a 5-point Likert scale which ranges from 1-5 (never/almost never to always/almost always) with higher scores indicating more prominent sensory features. A composite score of overall sensory features was calculated by combining results from the 97 quantitative items and was subsequently used in analyses.

1.3. Autism Features Composite Score. To create a composite score representative of individual differences in the prominence of autism-related features, data reduction via PCA was performed with the autism-feature caregiver-reported behavioral measures (total scores of the total raw SRS-2 scores, total raw SCQ scores, and total raw RBS-R scores [log-transformed for normality]). Scores collapsed onto a single factor (per scree plot of eigenvalues, BIC, and root mean squared error of approximation [RMSEA]) and the measures demonstrated similar sums of squares loadings onto the principal component: SRS-2= 0.89; SCQ = 0.73; RBS-R = 0.86.

References.

American Academy of Pediatrics & National Institute for Children's Health Quality (2002). NICHQ Vanderbilt Assessment Scales. Available at: https://www.nichq.org/sites/default/files/resource-file/NICHQ_Vanderbilt_Assessment_Scales.pdf [Accessed May 15, 2019].

Ausderau, K. K., Sideris, J., Little, L. M., Furlong, M., Bulluck, J. C., and Baranek, G. T. (2016). Sensory subtypes and associated outcomes in children with autism spectrum disorders: Sensory subtypes and outcomes in children with ASD. *Autism Research* 9, 1316–1327. doi: 10.1002/aur.1626.

Baranek, G. T. (2009). Sensory experiences questionnaire version 3.0. *Unpublished manuscript*.

Supplementary Table 1. Inclusion and exclusion criteria for intrahemispheric tractography in right and left hand networks

		Right Hand Network		Left Hand Network	
Network	Tract	Inclusion ROIs	Exclusion ROIs	Inclusion ROIs	Exclusion ROIs
Lateral Grasping	SMG-PMC	L SMG, L PMC	L SMG, L PMC, R Thalamus, brainstem	R SMG, R PMC	L SMG, L PMC, R Thalamus, brainstem
	PMC-M1	L PMC, L M1	L PMC, L M1, R Thalamus, brainstem	R PMC, R M1	L PMC, L M1, R Thalamus, brainstem
Corticospinal Output	CST	L M1, L inferior CST	L M1, L inferior CST, corpus callosum, R inferior POTPT	R M1, R inferior CST*	L M1, L inferior CST, corpus callosum, R inferior POTPT
Proprioception Input	VPL_{ThaL} S1	L VPL_{Thal}, L SI	L VPL_{Thal}, L SI, brainstem	R VPL_{Thal}, R SI	L VPL_{Thal}, L SI, brainstem
	S1-SMG	L S1, L SMG	L S1, L SMG, R thalamus, brainstem	R S1, L SMG	L S1, L SMG, R thalamus, brainstem
Cerebellar Modification	M1-PN	L M1, L inferior POTPT	L M1, L inferior POTPT, corpus callosum, R inferior CST	R M1, R inferior POTPT*	L M1, L inferior POTPT, corpus callosum, R inferior CST
	VL_{ThaL} M1	L VL_{Thal}, L M1	L VL_{Thal}, L M1, brainstem	R VL_{Thal}, R M1	L VL_{Thal}, L M1, brainstem

VPL, ventral posterolateral nucleus of the thalamus; S1, primary somatosensory cortex; SMG, supramarginal gyrus; PMC, premotor cortex; M1, motor cortex; PN, pontine nuclei; MCP, middle cerebellar peduncle; VL, ventrolateral nucleus of the thalamus; CST, corticospinal tract; POTPT, parietal occipital temporal pontine tract

Supplementary Table 2. Demographic information for participant sample with autistic group split by co-occurring ADHD features

	Non-Autistic	Autistic with elevated ADHD features	Autistic with reduced ADHD features	F value	*p*
	n=70	n=41	n=27		
Age(years), Mean(SD)	8.34(1.36)			.031	.96
Sex, % Female	34%	12%	30%	6.77	.03
Average Head Motion (AVD), Mean(SD)	0.53(0.42)	0.70(0.50)	0.61(0.47)	1.73	.18
Hand Preference, % Right-Handed	85.7%	20%	11%	3.62	.16
Maximum Grip, kg (Bilateral Avg), Mean(SD)	13.35(3.75)	13.27(4.37)	12.21(4.28)	.86	.42
Maximum Grip, kg (Right Hand), Mean(SD)	13.74(3.94)	13.58(4.58)	12.57(4.40)	.84	.43
Maximum Grip, kg (Left Hand), Mean(SD)	12.96(3.77)	12.96(4.36)	11.85(4.39)	.82	.44
IQ, Mean(SD)	114.41(12.14)	105.22(20.99)	108.74(16.08)	4.41	.01
SRS Total Score, Mean(SD)	21.94(13.55)	106.68(23.48	82.63(25.42	265.07	<.001
SCQ Total Score, Mean(SD)	1.45(1.88)	20.83(5.80)	18.0(7.12)	266.29	<.001
SEQ Total Score, Mean (SD)	138.5(21.3)	240.05(40.22)	203.19(37.93)	141.65	<.001
RBS-R Total Score, Mean(SD)	2.43(3.67)	40.27(25.48)	21.19(11.58)	82.24	<.001
NICHQ Total Score, Mean(SD)	9.4(6.0)	240.05(40.22)	203.19(37.93)	203.93	<.001
Taking Centrally Activating Medication, %	1%	47%	19%	32.8	<.001

^ Indicates χ^2 value instead of F-value

AVD Average Volume Displacement (Bastiani et al., 2019), SRS Social Responsiveness Scale (Constantino and Gruber, 2012), SCQ Social Communication Questionnaire (Rutter et al., 2003), RBS-R Repetitive Behavior Scale-Revised (Bodfish et al., 2000; Lam & Aman, 2007), NICHQ National Initiative for Children's Healthcare Quality Vanderbilt Assessment Scales (American Academy of Pediatrics & National Institute for Children's Health Quality, 2002), SEQ Sensory Experience Questionnaire (Baranek, 2009)

Supplementary Table 3. Within-tracts post hoc analyses predicting structural properties from the interaction between maximal grip strength and diagnosis (autistic vs non-autistic)

Metric of Interest	Tract of Interest	b	SE	95% Confidence Interval		*t*	*p* (FDR-adjusted)
				Lower	Upper		
R1							
	ML	0.000	0.001	-0.002	0.002	-0.33	.83
	VPL Thal -S1	0.001	0.002	-0.004	0.006	0.43	.83
	S1 -SMG	0.001	0.002	-0.002	0.005	0.62	.83
	SMG-PMC	0.000	0.003	-0.007	0.006	-0.13	.90
	PMC -M1	-0.007	0.003	-0.012	-0.001	-2.42	.17
	M1-PN	-0.003	0.002	-0.007	0.000	-1.74	.42
	MCP	0.001	0.001	-0.002	0.004	0.80	.83
	SCPCT	-0.001	0.001	-0.003	0.002	-0.46	.83
	VL Thal -M1	-0.001	0.002	-0.006	0.003	-0.49	.83
	CST	-0.002	0.002	-0.005	0.001	-1.30	.66

All post hoc analyses controlled for age, and sex

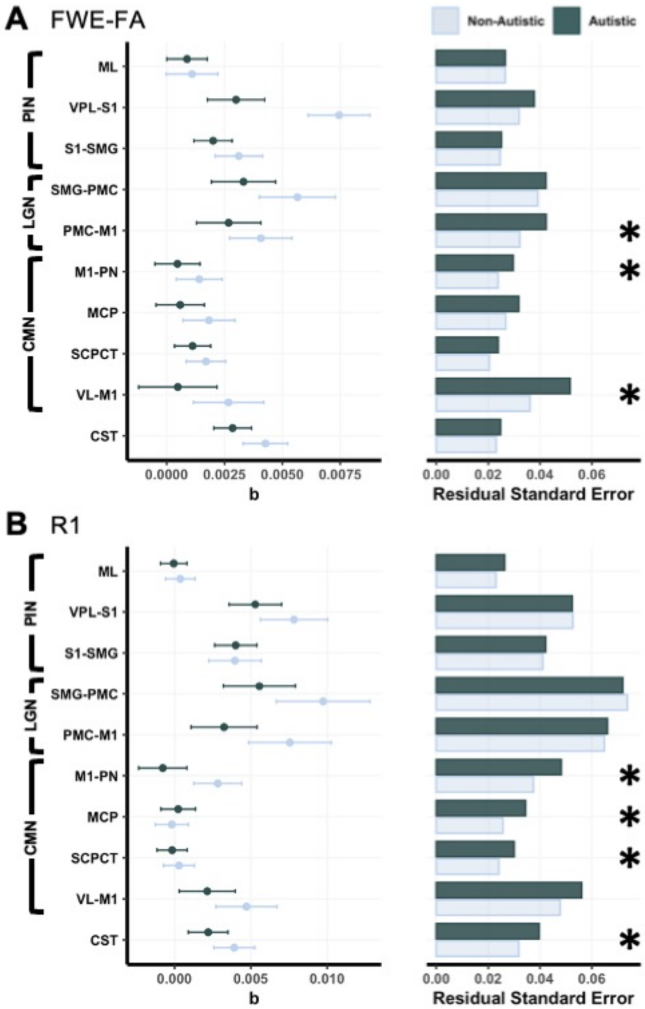

Supplementary Figure 1. Autistic vs non-autistic population model comparison. Comparisons of b values (+/- standard error) and residual standard error of models predicting A) FWE-FA and B) R1 of sensorimotor tracts of interest in autistic and non-autistic children. Asterisk (*) represents models that show significantly higher variance in the autistic population compared to the non-autistic population. PIN, proprioception input network; LGN, lateral grasping network; CMN, cerebellar modification network

Supplementary Table 4. Results of F tests to compare variance of models predicting sensorimotor network microstructure from grip strength in autistic vs non-autistic children.

Metric of Interest	Tract of Interest	F	Df num	Df den	p
FWE-FA					
	ML	1.00	63	65	.50
	VPL Thal -S1	1.39	63	65	.10
	S1 -SMG	1.06	63	65	.41
	SMG-PMC	0.43	63	65	>.99
	PMC -M1	1.73	63	65	.02
	M1-PN	1.56	63	65	.04
	MCP	1.41	63	65	.05
	SCPCT	1.36	63	65	.11
	VL Thal -M1	2.03	63	65	<.001
	CST	1.16	63	65	.28
R1					
	ML	1.33	63	65	.13
	VPL Thal -S1	0.99	63	65	.51
	S1-SMG	1.05	63	65	.42
	PMC-M1	0.95	63	65	.58
	SMG-PMC	1.04	63	65	.44
	M1-PN	1.65	63	65	.02
	MCP	1.78	63	65	.01
	SCPCT	1.56	63	65	.04
	VL Thal-M1	1.38	63	65	.10
	CST	1.55	63	65	.04

All post hoc analyses controlled for age, and sex
FWE-FA analyses additionally controlled for head motion during the DWI scan

Supplementary Table 5. Results of linear mixed effect models predicting sensorimotor network microstructure from sensory features and maximum grip strength in autistic children

Metric of Interest	Predictor	Sum of Squares	Mean Square	F	P
FWE-FA					
	Grip Strength	0.003	0.003	2.46	.12
	Sensory Features	0.001	0.001	1.16	.29
	Sensorimotor Tract	0.114	0.013	12.35	<.001
	Age	0.008	0.008	7.53	.01
	Sex	0.000	0.000	0.13	.72
	Head Motion	0.023	0.023	22.75	<.001
	Grip Strength x Sensory Features	0.005	0.005	4.58	.04
	Grip Strength x Tract	0.018	0.002	1.99	.04
	Sensory Features x Tract	0.004	0.000	0.43	.92
	Grip Strength x Sensory Features x Tract	0.018	0.002	1.96	.06
R1					
	Grip Strength	0.003	0.003	1.68	.20
	Sensory Features	0.000	0.000	0.01	.91
	Sensorimotor Tract	0.071	0.008	4.78	<.001
	Age	0.005	0.005	3.13	.08
	Sex	0.000	0.000	0.21	.64
	Grip Strength x Sensory Features	0.005	0.005	2.98	.09
	Grip Strength x Tract	0.021	0.002	1.43	.17
	Sensory Features x Tract	0.008	0.001	0.51	.87
	Grip Strength x Sensory Features x Tract	0.019	0.002	1.30	.23

Supplementary Table 6. Results of linear mixed effect models predicting sensorimotor network microstructure from IQ and maximum grip strength in autistic children

Metric of Interest	Predictor	Sum of Squares	Mean Square	F	p
FWE-FA					
	Grip Strength	0.0009	0.0009	0.82	.37
	IQ	0.0001	0.0001	0.14	.71
	Sensorimotor Tract	0.1234	0.0137	13.12	<.001
	Age	0.0104	0.0104	9.96	<.001
	Sex	0.0014	0.0014	1.30	.26
	Head Motion	0.0179	0.0179	17.11	<.001
	Grip Strength x IQ	0.0003	0.0003	0.24	.63
	Grip Strength x Tract	0.0069	0.0008	0.73	.68
	IQ x Tract	0.0015	0.0002	0.16	>.99
	Grip Strength x IQ x Tract	0.0060	0.0007	0.64	.76
R1					
	Grip Strength	0.0060	0.0060	3.60	.06
	IQ	0.0045	0.0045	2.68	.11
	Sensorimotor Tract	0.0696	0.0077	4.61	<.001
	Age	0.0031	0.0031	1.84	.18
	Sex	0.0003	0.0003	0.19	.66
	Grip Strength x IQ	0.0029	0.0029	1.74	.19
	Grip Strength x Tract	0.0117	0.0013	0.78	.64
	IQ x Tract	0.0080	0.0009	0.53	.85
	Grip Strength x IQ x Tract	0.0067	0.0007	0.44	.91

Supplementary Table 7. Results of linear mixed effect models predicting sensorimotor network microstructure from autism features and maximum grip strength in autistic children

Metric of Interest	Predictor	Sum of Squares	Mean Square	F	P
FWE-FA					
	Grip Strength	0.006	0.006	6.12	.02
	Autism Features	0.002	0.002	1.65	.20
	Sensorimotor Tract	3.683	0.409	405.97	<.001
	Age	0.008	0.008	7.66	.01
	Sex	0.001	0.001	0.71	.40
	Head Motion	0.014	0.014	14.19	<.001
	Grip Strength x Autism Features	0.005	0.005	4.85	.03
	Grip Strength x Tract	0.021	0.002	2.27	.02
	Autism Features x Tract	0.008	0.001	0.90	.53
	Grip Strength x Autism Features x Tract	0.021	0.002	2.37	.01
R1					
	Grip Strength	0.007	0.007	4.10	.05
	Autism Features	0.000	0.000	0.11	.74
	Sensorimotor Tract	1.612	0.179	107.60	<.001
	Age	0.005	0.005	3.09	.08
	Sex	0.000	0.000	0.18	.68
	Grip Strength x Autism Features	0.002	0.002	0.97	.33
	Grip Strength x Tract	0.076	0.008	5.04	<.001
	Autism Features x Tract	0.003	0.000	0.19	.99
	Grip Strength x Autism Features x Tract	0.015	0.002	0.99	.45

Supplementary Table 8. Results of within-tracts post hoc analyses predicting structural properties from the maximal grip strength-by-autism features interaction in autistic children

Metric of Interest	Tract of Interest	b	SE	95% Confidence Interval Lower	95% Confidence Interval Upper	t	p (FDR-adjusted)
FWE-FA							
	ML	-0.0001	0.0009	-0.002	0.002	1.01	.89
	VPL Thal -S1	0.0021	0.0013	-0.001	0.005	2.42	.25
	S1 -SMG	0.0012	0.0009	-0.001	0.003	2.43	.30
	SMG-PMC	0.0023	0.0015	-0.001	0.005	2.40	.25
	PMC -M1	0.0026	0.0015	0.000	0.006	1.93	.25
	M1-PN	0.0017	0.0010	0.000	0.004	0.48	.25
	MCP	0.0007	0.0011	-0.002	0.003	0.56	.67
	SCPCT	0.0007	0.0008	-0.001	0.002	1.43	.58
	VL Thal -M1	0.0027	0.0018	-0.001	0.006	0.29	.25
	CST	0.0002	0.0009	-0.002	0.002	3.52	.88

All post hoc analyses controlled for age, sex, head motion during the DWI scan, and main effects of grip strength and autism features

Supplementary Table 9. Results of linear mixed effect models predicting sensorimotor network microstructure from medication status and maximum grip strength in autistic children

Metric of Interest	Predictor	Sum of Squares	Mean Square	F	p
FWE-FA					
	Grip Strength	0.0032	0.0032	3.19	.08
	Medication Status	0.0000	0.0000	0.03	.86
	Sensorimotor Tract	2.3621	0.2625	259.14	<.001
	Age	0.0047	0.0047	4.67	.03
	Sex	0.0014	0.0014	1.34	.25
	Head Motion	0.0254	0.0254	25.03	<.001
	Grip Strength x Medication Status	0.0001	0.0001	0.05	.82
	Grip Strength x Tract	0.0110	0.0012	1.21	.29
	Medication Status x Tract	0.0171	0.0019	1.88	.05
	Grip Strength x Medication Status x Tract	0.0100	0.0011	1.10	.36
R1					
	Grip Strength	0.0025	0.0025	1.49	.23
	Medication Status	0.0100	0.0100	6.06	.02
	Sensorimotor Tract	0.9643	0.1071	65.08	<.001
	Age	0.0026	0.0026	1.59	.21
	Sex	0.0002	0.0002	0.10	.75
	Grip Strength x Medication Status	0.0002	0.0002	0.13	.72
	Grip Strength x Tract	0.0433	0.0048	2.92	.002
	Medication Status x Tract	0.0148	0.0016	1.00	.44
	Grip Strength x Medication Status x Tract	0.0144	0.0016	0.97	.46
